# A Simulation Study of a Bandpass Filter Formed by CNT-Core Cu-TSVs with Enhanced Thermal Management

**DOI:** 10.3390/mi17060724

**Published:** 2026-06-15

**Authors:** Han Wang, Yingtao Ding, Ziyue Zhang, Jiaxuan Zhang, Anda Zhang, Xiang Pei, Zhiming Chen

**Affiliations:** School of Integrated Circuits and Electronics, Beijing Institute of Technology, Beijing 100081, China; 3220215100@bit.edu.cn (H.W.); ytd@bit.edu.cn (Y.D.); 3120256134@bit.edu.cn (J.Z.); 3220231951@bit.edu.cn (A.Z.); 18347156373@163.com (X.P.); czm@bit.edu.cn (Z.C.)

**Keywords:** bandpass filter, through-silicon-via (TSV), carbon nanotubes (CNTs), thermal management

## Abstract

Bandpass filters based on through-silicon-via (TSV) interposers offer advantages such as compact footprint, excellent radio frequency (RF) performance, simplified processing, and low cost. However, as power densities in three-dimensional (3D) integrated circuits continue to rise, thermal management has become a critical performance bottleneck. In this work, we present a TSV-based bandpass filter design where the TSVs feature annular Cu conductors with carbon nanotube (CNT) cores. The annular Cu structure provides the required vertical electrical connectivity, while the high-thermal-conductivity CNT core facilitates inter-layer heat dissipation. RF simulations confirm that the RF characteristics of the filter remain comparable to those of filters based on conventional TSVs with Cu-pillar conductors or TSVs with annular Cu conductors and polymer cores such as benzocyclobutene (BCB). In addition, multiphysics simulations demonstrate that the proposed filter exhibits a maximum steady-state temperature of only 89.1 °C with a 5 W constant heat source attached to the interposer surface and a heat sink at the bottom side, presenting an efficient reduction compared to the other two types. The filter also shows reduced thermally induced surface deformation, confirming the thermal benefits of the CNT cores. Furthermore, comprehensive parametric analyses involving the influences of critical TSV structural parameters on the TSV-based capacitors and inductors are performed, providing guidelines for customized filter design. We believe the proposed design highlights a promising pathway for addressing the thermal management challenges in high-density RF integrated microsystems.

## 1. Introduction

As semiconductor manufacturing processes approach their physical limits, the scaling-driven performance enhancement predicted by Moore’s Law faces severe challenges [1,2,3]. Through-silicon-via (TSV) technology, which establishes vertical interconnects between stacked chips, has emerged as a core technology for three-dimensional (3D) integration, offering shortened interconnect lengths and reduced power consumption, thereby extending Moore’s Law [3,4,5,6]. Beyond serving as interconnects, TSVs can be integrated with re-distribution layers (RDLs) to form functional passive components such as capacitors, inductors, and filters, leveraging their unique structural and electrical properties [7,8,9,10]. Such TSV-based devices typically offer advantages in device size, electrical performance, fabrication cost, and design flexibility, making them attractive for high-density heterogeneous integrated microsystems.

However, with the continuous rise in 3D integration density, heat dissipation per unit volume increases sharply, making thermal management a primary obstacle to system performance and reliability [8,11,12]. In heterogeneous integrated systems employing TSVs, effective heat dissipation between layers is critical to avoid localized heat accumulation [13,14]. This challenge is particularly acute for thick interposers, where large-dimension TSVs are required. Fully filled Cu-pillar conductors are not suitable in such cases for two reasons. On the one hand, complete Cu filling of large vias becomes increasingly difficult, expensive, and time-consuming. On the other hand, the significant coefficient of thermal expansion (CTE) mismatch between Cu and Si induces substantial thermal stress during thermal treatment or high-temperature operation, potentially causing TSV extrusion, liner cracking, or chip delamination [11,14,15,16]. To mitigate fabrication difficulties and reduce thermal stress in large-dimension TSVs, annular Cu conductors with polymer cores such as benzocyclobutene (BCB) are widely adopted [17,18,19]. However, the low thermal conductivity of BCB could impede inter-layer heat dissipation in the integrated systems, exacerbating thermal accumulation.

Recently, a novel design employing carbon nanotubes (CNTs) as the central core in large-dimension TSVs has been proposed to enhance the thermal dissipation between stacked layers [20]. Apart from acting as TSV conductors [21], the CNTs are considered ideal candidates for improving internal thermal management of composite TSVs due to their exceptional axial thermal conductivity and excellent electrical ballistic transport properties [22,23,24]. Furthermore, CNTs exhibit an extremely low CTE that is highly compatible with the Si substrate, alleviating thermo-mechanical reliability concerns [24,25,26]. In terms of practical implementation, CNTs can be formed in TSVs by methods like direct growth, transfer, and vacuum-assisted filling [20,26,27,28,29,30,31]. Although the thermal benefits of CNT cores have been demonstrated, investigations on the possible impacts of integrating CNT cores on the electrical characteristics of TSV-based devices and the combined thermal behavior of such devices containing both TSVs and RDLs are still lacking.

By incorporating the CNT-core Cu-TSV structure into a TSV-based bandpass filter design, this work presents an electrical–thermal co-designed bandpass filter that combines the benefits of common TSV-based radio frequency (RF) integrated passive devices (IPDs) with enhanced thermal performance. The possible influences of central core materials on the RF performance of IPDs are evaluated using Ansys HFSS 15.0 electromagnetic simulations. And the thermal characteristics of filters with different TSV configurations including inter-layer heat dissipation and thermally induced deformation are analyzed by COMSOL Multiphysics 6.3 simulations. Additionally, to provide comprehensive design guidelines on IPDs using CNT-core Cu-TSVs, the effects of key structural parameters on TSV-based capacitors and inductors are systematically investigated.

This paper is organized as follows: Section 2 presents the design of a bandpass filter and its physical implementation by using TSVs and RDLs in HFSS modeling. Section 3 compares the RF performance of IPDs using different TSV configurations. Section 4 demonstrates the thermal performance improvements enabled by CNT-core TSVs. Section 5 investigates the influences of key structural parameters on TSV-based capacitors and inductors to provide design guidelines. Finally, Section 6 concludes the work.

## 2. Design of a TSV-Based Bandpass Filter

**Figure 1:**
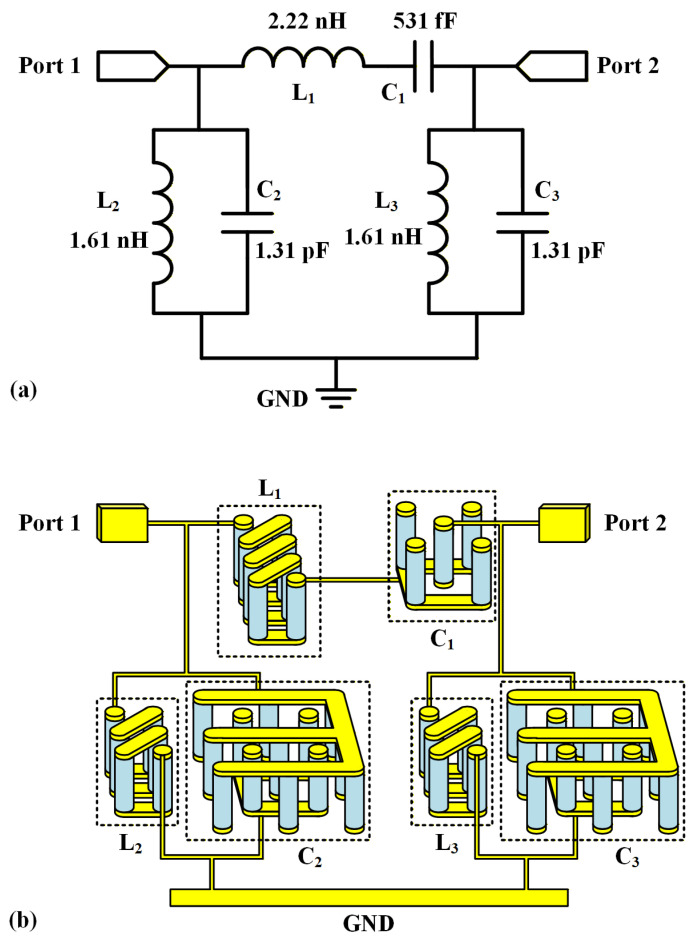
Filter design: (**a**) circuit model with specific capacitances and inductances, (**b**) physical model with capacitors and inductors formed by TSVs and RDLs.

Assisted by the Keysight ADS 2020 circuit simulator, a third-order Butterworth bandpass filter with central frequency of 2.4 GHz is designed as an implementation example, a frequency that is commonly used in WLAN applications. As shown in Figure 1a, this filter comprises three capacitors and three inductors with specific values. In this work, these components are realized using topological TSV-based coaxial-like capacitors and solenoid inductors, as illustrated in Figure 1b. To achieve the targeted capacitances and inductances, the structural parameters such as TSV diameter, TSV height, pitch between adjacent TSVs, and numbers of utilized TSVs are carefully designed following established guidelines [10,32] and validated in Ansys HFSS. Taking the capacitor component C_2_ as an example, it consists of 13 identical TSVs arranged in three tiers, where the central TSV (first tier) and the eight outer TSVs (third tier) are connected via front-side RDLs while the four intermediate TSVs (second tier) are connected via back-side RDLs. The TSVs have a diameter of 50 μm, a height of 500 μm, and a 2 μm thick insulating liner. The pitch between adjacent outer TSVs is $60\sqrt{2}$ μm, resulting in an inner–outer pitch of 60 μm. Note that the TSV conductors are modeled as fully filled Cu pillars, and parylene is chosen as the liner material for its low dielectric constant, good dielectric properties, and superior conformality [33,34,35].

This filter design exhibits several key features. First, the TSV-based coaxial-like capacitors leverage the parasitic capacitance between adjacent TSV tiers. Second, the TSV-based solenoid inductors utilize pairs of adjacent TSVs and connecting RDLs to form compact coil structures, avoiding the large lateral footprint of planar inductors. Third, because all components are constructed from identical TSVs and RDLs, this filter design eliminates the need for external discrete components and overcomes the area limitations of conventional planar filters. Finally, the utilization of vertical space allows the filter to be embedded within the interposer substrate, thereby releasing more surface areas for integrating other chips and maximizing the functional density per unit volume.

## 3. RF Performance Comparison of IPDs Based on Three TSV Configurations

**Figure 2:**
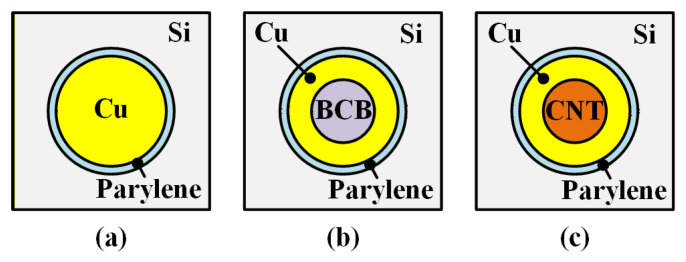
Schematics of different TSV configurations: (**a**) Cu-pillar TSV, (**b**) BCB-core TSV with annular Cu conductor, and (**c**) CNT-core TSV with annular Cu conductor.

To evaluate the possible influences of substituting Cu-pillar conductors with annular Cu conductors and CNT cores on the RF performance of TSV-based IPDs, full-wave electromagnetic simulations are carried out using Ansys HFSS. Three TSV configurations are compared, as shown in Figure 2, where the liners are parylene and inner fillers are, respectively, fully filled Cu pillar (Figure 2a), annular Cu conductor with BCB core (Figure 2b), and annular Cu conductor with CNT core (Figure 2c). Notably, the physical fabrication process of such CNT-core TSVs has already been successfully realized [20]. The filter shown in Figure 1b, along with the capacitor component C_2_ and inductor component L_2_, are simulated for comparison under identical structural parameters except for the core material, ensuring that differences arise solely from filler properties. Note that the CNT core formed by abundant CNT bundles is treated as an equivalent bulk material in the simulations with a typical conductivity of 2.7 × 10^7^ S/m [36,37]. The diameter of the central core is 20 μm.

**Figure 3:**
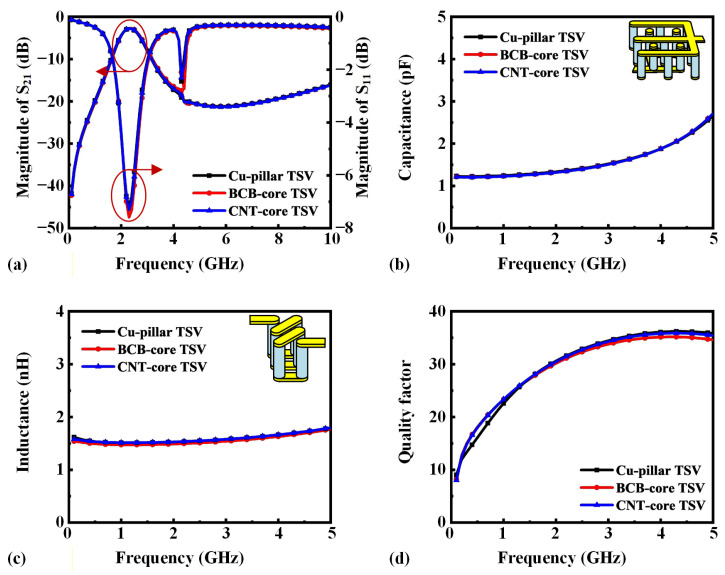
RF performance comparison of IPDs based on different TSV configurations: (**a**) S-parameters of the TSV-based filters, (**b**) capacitance values of the TSV-based capacitors (C_2_), (**c**,**d**) inductance values and quality factors of the TSV-based inductors (L_2_). The black, red, and blue curves correspond to Cu-pillar, BCB-core, and CNT-core TSVs, respectively. The capacitors and inductors used in the comparisons are shown in the insets.

As illustrated in Figure 3a, the S-parameters of the three filters based on different TSV configurations are nearly identical across 0.1–10 GHz. Figure 3b presents the capacitance–frequency curves of the C_2_ capacitors, as shown in the inset, based on the three TSV configurations in the 0.1–5 GHz band; Figure 3c,d similarly give the three inductance– and quality factor–frequency curves of the L_2_ inductors, as shown in the inset of Figure 3c. It can be seen from these simulation results that both the capacitors and inductors exhibit nearly identical performance for all three TSV configurations. This can be explained as follows: (1) The capacitance of the TSV-based coaxial-like capacitor mainly originates from the parasitic capacitances between TSVs from adjacent tiers, which are determined by the outer radii of the Cu conductors and the pitches between inner and outer TSVs, thus not being influenced by the filler materials. (2) The performance of the TSV-based inductor could be primarily attributed to the skin effect in high-frequency signal transmission, where the current density concentrates predominantly near the conductor surfaces of TSVs as the frequency increases, while the current distributions in the central regions are negligible. Consequently, whether the TSV centers are filled with the insulating BCB polymer or the conductive CNTs, there are actually few impacts on the equivalent impedance and insertion loss of the macroscopic signal transmission path; thus, the inductor performance is not influenced. (3) As the capacitors and inductors are not changed with the TSV central fillers, the constructed filters also show almost identical performance for different TSV configurations. These results confirm that incorporating CNTs as the central cores in TSVs with annular Cu conductors preserves the RF performance of TSV-based IPDs with fully filled Cu-pillar TSVs, while offering potential thermal benefits.

## 4. Thermal Performance Comparison of IPDs Based on Three TSV Configurations

**Figure 4:**
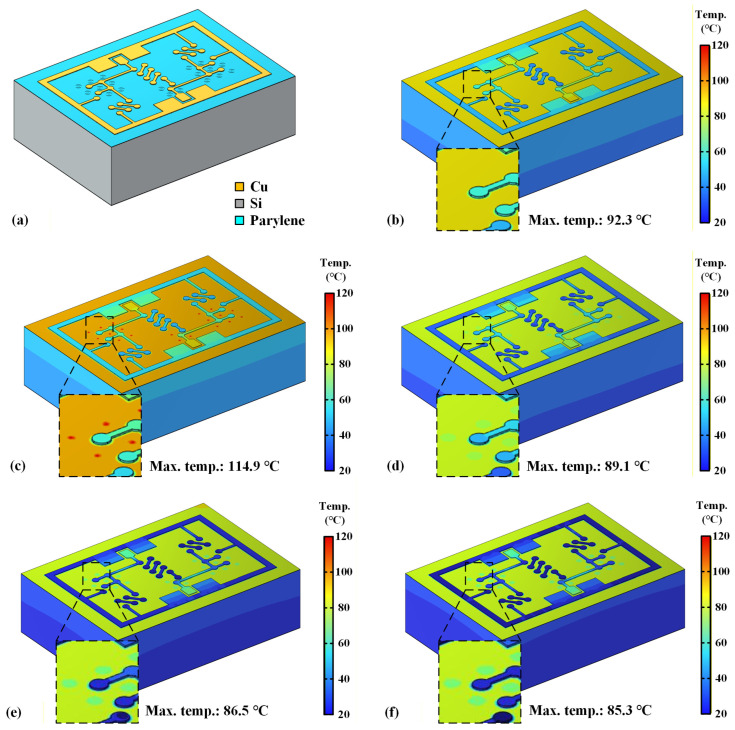
Evaluation of heat dissipation performance of filters based on different TSV configurations: (**a**) initial model without heating, (**b**–**d**) steady-state temperature distributions of filters based on Cu-pillar, BCB-core, and CNT-core TSVs under stable heating, respectively. (**e**,**f**) are results for filters based on CNT-TSVs with CNT-core diameters of 30 and 40 μm, respectively.

To validate the thermal management benefits of CNT-core TSVs, multiphysics thermal simulations are conducted on IPDs based on these three TSV configurations including Cu-pillar, BCB-core, and CNT-core TSVs using COMSOL. The initial filter model built in COMSOL is given in Figure 4a. Considering that the thermal conductivity of CNTs varies with parameters like growth orientation and filling factor of the bundles, a typical value of 1750 W/(m·K) is first set in this work to provide a moderate evaluation and the CNT core is assumed as a bulk and isotropic material with an elastic modulus of 1 TPa and Poisson’s ratio of 0.35, similar to the reported literature [38,39,40,41]. In the simulations, a 5 W constant heat source is applied to the top surface of the interposer containing the TSV-based filter for each TSV configuration to simulate realistic operating conditions of long-term device operation, and a constant temperature boundary condition of 20 °C is set at the bottom surface to represent an ideal heat sink environment.

**Figure 5:**
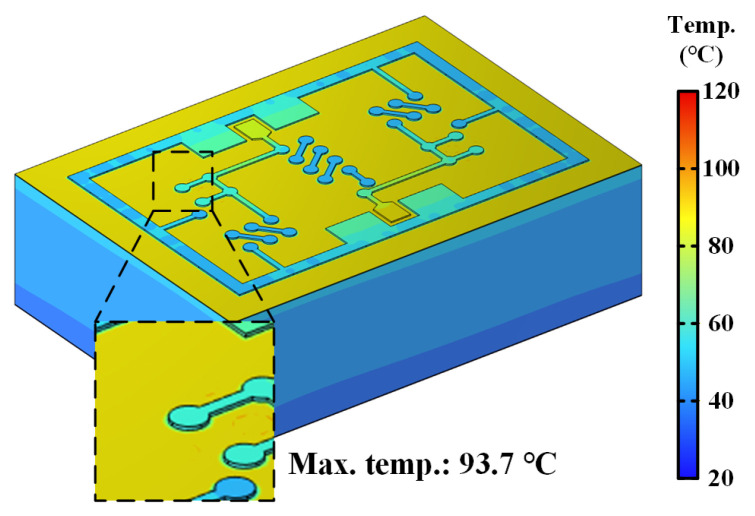
Evaluation of heat dissipation performance of filter based on CNT-core TSVs with CNT thermal conductivity of 100 W/m·K.

Figure 4b–d present the steady-state temperature distributions of filters based on Cu-pillar, BCB-core, and CNT-core TSVs under stable heating, respectively, which clearly reveal the significant impact of filler material properties on the thermal field distribution. And the highest temperatures of each component in these filters are listed in Table 1. As shown in Figure 4b, the filter based on conventional Cu-pillar TSVs presents a maximum temperature of 92.3 °C. However, as aforementioned, such Cu-pillar TSVs could lead to fabrication difficulties and reliability issues. By replacing the fully filled Cu-pillar conductor with annular Cu conductor and central BCB core, these issues can be alleviated to a certain degree. However, the maximum temperature of the filter based on BCB-core TSVs reaches 114.9 °C according to Figure 4c, and the high-temperature regions are highly concentrated within the BCB-filled central areas of the TSVs, confirming that the BCB cores are poor in heat dissipation between layers. In contrast, Figure 4d highlights the superior heat dissipation performance of the CNT-core TSVs, where the maximum temperature of the filter is only 89.1 °C, representing a reduction of 25.8 °C compared to the BCB-core one and even a small reduction of 3.2 °C compared to the Cu-pillar one. As shown in Table 1, such improvement in heat dissipation performance is reflected homogeneously on each inductor and capacitor component. This improvement is attributed to the extremely high axial thermal conductivity of CNTs, which act as “thermal highways” to efficiently channel generated heat toward the heat sink. The fully filled Cu-pillar TSV exhibits a slightly weaker heat dissipation performance due to the internal metal lattice scattering and its thermal conductivity. Note that although the CNT-core TSV shows a small thermal gain over Cu-pillar TSV, it is advantageous in terms of lower process cost, process difficulty, and higher reliability. Conversely, the BCB-core TSV displays the worst heat dissipation performance, which is due to the excessive thermal resistance of BCB that impedes outward heat dissipation and is prone to causing localized thermal accumulation. Notably, in the CNT-core configuration, the maximum temperature point shifts from the TSV center to the surface parylene insulation layer region. This phenomenon strongly demonstrates that the CNTs act as efficient thermal conduction paths where the internal thermal resistance of the TSV is significantly lower than that of the external insulation layer, rendering the TSV filler itself no longer a thermal bottleneck. Such alteration in thermal distribution facilitates further temperature reduction through the optimization of external cooling methods, showcasing its potential in high-heat-flux packaging. Furthermore, another two filters formed by CNT-TSVs with different CNT-core diameters are simulated in Figure 4e,f, and the maximum temperatures of each component are summarized in Table 1. It can be seen that a larger CNT-core diameter contributes to further enhancing the heat dissipation performance, which is consistent with previous analyses. In practice, the fabricated CNT core could have effective thermal conductivity much lower than the theoretical one (for example, in the range of 100–400 W/(m·K)); therefore, another simulation on the heat dissipation performance of the filter based on CNT-core TSVs with CNT thermal conductivity of 100 W/(m·K) is carried out. As shown in Figure 5, although the thermal performance is lower compared to the case with CNT thermal conductivity of 1750 W/(m·K) in Figure 4d, the thermal benefit over the filter based on BCB-core TSVs in Figure 4c is still remarkable; for instance, the highest temperature shows a shrinkage of 21.2 °C. In addition, it is noteworthy that the heat dissipation performance of the filter based on CNT-core TSVs is comparable to that of the one based on Cu-pillar TSVs shown in Figure 4b, despite a much lower CNT thermal conductivity being used, highlighting its improved thermal performance compared to the one based on BCB-core TSVs while maintaining advantages such as lower Cu electroplating difficulty and cost, and higher thermo-mechanical reliability compared to the one based on Cu-pillar TSVs.

**Figure 6:**
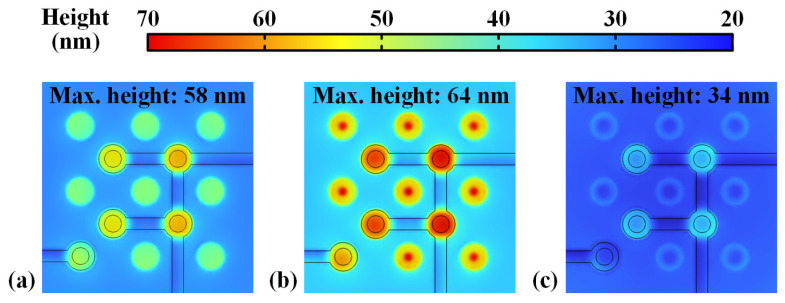
Evaluation of thermally induced deformation of filters based on different TSV configurations: (**a**) Cu-pillar TSVs, (**b**) BCB-core TSVs, and (**c**) CNT-core TSVs. The C_2_ regions are shown for a clearer observation.

**Figure 7:**
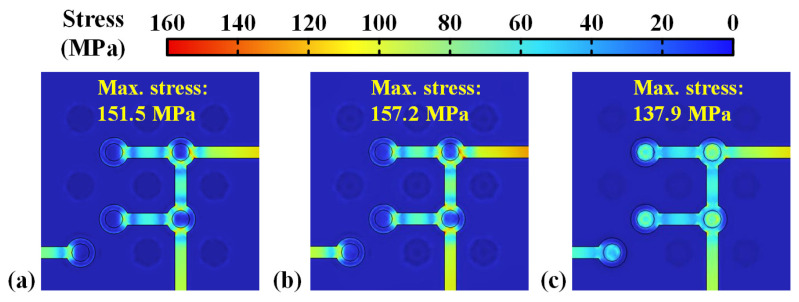
Evaluation of thermo-mechanical stress distributions of filters based on different TSV configurations: (**a**) Cu-pillar TSVs, (**b**) BCB-core TSVs, and (**c**) CNT-core TSVs. The C_2_ regions are shown for a clearer observation.

Figure 6 shows the thermally induced deformation profiles of the enlarged C_2_ regions for the filters based on three TSV configurations. The heating condition is the same as the heat dissipation simulations. It can be seen that the maximum deformation heights for these three devices are 58 nm, 64 nm, and 34 nm, respectively, presenting similar trends with the heat dissipation simulation results. Thanks to the low CTE of CNTs and the low local temperature, the filter based on CNT-core TSVs has the lowest thermally induced deformation, which further validates the significant enhancement of CNT-cores on the thermal performance of the TSV-based IPDs. Similarly, Figure 7 presents the thermo-mechanical stress distributions of the enlarged C_2_ regions for these three filters. It can be seen that the CNT-core TSVs also contributes to reducing the thermo-mechanical stresses, thus further enhancing device reliability.

## 5. Parametric Analyses of Capacitors and Inductors Based on CNT-Core TSVs

To accommodate the diverse requirements of future communication standards, systematic parametric analyses of the key structural parameters of capacitors and inductors based on CNT-core TSVs are carried out. Note that these analyzing results provide a foundation for designing TSV-based filters with varied characteristics by tuning individual capacitor and inductor components. The capacitor C_2_ and inductor L_2_ are used as representative elements in the parametric study.

**Figure 8:**
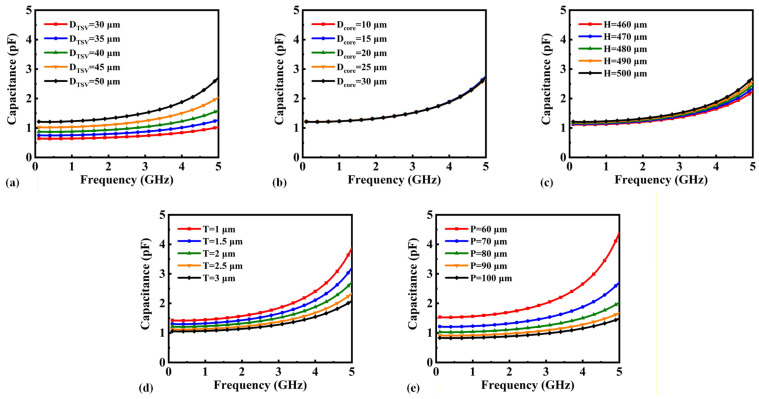
Influences of structural parameters on the capacitance of the TSV-based capacitor (C_2_): (**a**) TSV diameter (D_TSV_), (**b**) CNT-core diameter (D_core_), (**c**) TSV height (H), (**d**) liner thickness (T), and (**e**) pitch between inner and outer TSVs from adjacent TSV tiers (P).

Figure 8 illustrates how the capacitance of C_2_ varies with critical structural parameters. Figure 8a shows that increasing the TSV diameter enlarges the effective overlapping area of the capacitor, resulting in a nearly linear increase in capacitance across the simulated frequency range. Figure 8b confirms that, for a fixed TSV diameter, varying the CNT-core diameter does not affect capacitance, consistent with the earlier discussion of Figure 3b. Similarly, as shown in Figure 8c, increasing the TSV height expands the capacitive area, thereby raising capacitance. Furthermore, as evidenced in Figure 8d,e, both the thickness of the insulating liner and the pitch between the inner and outer TSV tiers significantly influence capacitance. According to the operating principle of TSV-based capacitors, reducing the liner thickness or narrowing the inter-tier spacing effectively decreases the separation between the conductive plates. This reduction leads to a substantial increase in both the TSV parasitic capacitance and the substrate coupling capacitance, thereby raising the total capacitance. These results suggest that, for a fixed layout, adjusting the liner thickness via the parylene-deposition process offers an effective means of tuning capacitance without requiring major modifications to the TSV dimensions. Moreover, increasing the number of surrounding TSVs or adding more tiers can multiplicatively enhance the capacitance of the coaxial-like structure.

**Figure 9:**
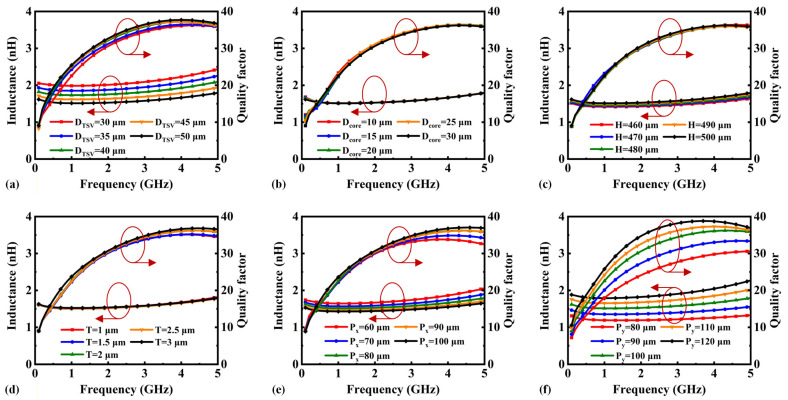
Influences of structural parameters on the inductance and quality factor of the TSV-based inductor (L_2_): (**a**) TSV diameter (D_TSV_), (**b**) CNT-core diameter (D_core_), (**c**) TSV height (H), (**d**) liner thickness (T), (**e**,**f**) pitches between two adjacent TSVs in the x- and y-directions (P_x_ and P_y_), respectively, where the y-direction is defined as the direction in which the two adjacent TSVs are connected by an RDL, whereas the two adjacent TSVs are not directly connected in the x-direction.

For the inductor component, two adjacent TSVs in the y-direction, together with the connecting RDLs, form an equivalent coil. The equivalent cross-sectional area of the inductor is determined by the spacing between these two TSVs and the TSV height. The spacing between two adjacent TSVs in the x-direction where these TSVs are not directly connected defines the loop pitch of the inductor. Figure 9 plots the impact of various structural parameters on the inductance and quality factor of the inductor L_2_. As shown in Figure 9a, increasing the TSV diameter reduces the effective cross-sectional area of the inductor, which decreases magnetic flux and consequently lowers inductance. At the same time, the resistance decreases, and conductor losses due to the skin effect are reduced at high frequencies, improving the quality factor. Figure 9b indicates that, similar to the capacitor, varying the CNT-core diameter has a negligible effect on inductor performance for a fixed TSV diameter. Figure 9c shows that increasing the TSV height expands the inductor’s cross-sectional area, enhancing magnetic flux and raising inductance. The accompanying increase in substrate thickness also yields a more pronounced increase in high-frequency impedance relative to the resistance, thereby improving the quality factor. Figure 9d illustrates that variations in parylene thickness have minimal impact on both inductance and quality factor, indicating that the magnetic field is primarily distributed in the surrounding Si substrate and is largely unaffected by the thin dielectric layer. Regarding array-spacing effects, Figure 9e shows that increasing the pitch in the x-direction (P_x_) reduces mutual inductance, slightly lowering total inductance, while the reduced proximity effect slightly decreases resistance; these opposing trends leave the quality factor largely unchanged. Figure 9f demonstrates that increasing the pitch in the y-direction (P_y_) enlarges the coil’s cross-sectional area, increasing magnetic flux and thereby raising inductance and improving the quality factor.

In summary, as the CNT-core diameter has few effects on the RF performance of the IPDs, the TSV dimensions including TSV diameter, TSV height, liner thickness, and TSV pitch can be first engineered to fit specific RF requirements in practical applications. And then the CNT-core diameter could be relatively larger to obtain higher heat dissipation performance. Note that the thickness of the annular Cu conductor cannot be too thin to guarantee enough electrical transmission capability and good manufacturing stability.

## 6. Conclusions

In this work, we present the design and validation of a bandpass filter based on TSVs with CNT cores. Multiphysics simulations confirm that the CNT-core TSVs establish efficient vertical thermal pathways, which can be attributed to the ultra-high thermal conductivity of CNTs. This reduces the steady-state maximum temperature of the TSV-based filter under a constant heat source to 89.1 °C, lower than the temperatures observed for both Cu-pillar and BCB-core TSV configurations. Simultaneously, the location of the maximum temperature shifts from the internal TSV center to the surface parylene insulation region, creating room for further system-level thermal optimization. Additionally, the thermally induced deformation is also reduced in the filter based on CNT-core TSVs. Electromagnetic simulations demonstrate that replacing conventional Cu-pillar TSVs with CNT-core TSVs does not degrade the RF performance of the IPDs. Furthermore, systematic parametric analyses provide clear guidelines for adjusting the characteristics of TSV-based capacitors and inductors, which can be directly applied to the design of tailored TSV-based filters. In summary, the use of CNT-core TSVs with annular Cu conductors brings in benefits including simplifying the electroplating of Cu conductors and improving the thermo-mechanical reliability due to the use of annular Cu conductors, as well as enhancing the overall thermal performance in terms of heat dissipation, thermally induced deformation, and thermo-mechanical stresses by further replacing the BCB fillers with CNT fillers. Consequently, the filter based on CNT-core TSVs combines superior heat dissipation capability with reliable RF performance, achieving a synergistic improvement in thermo-electrical performance. This approach offers a promising and effective solution for addressing both signal integrity and thermal management challenges in next-generation high-density 3D integrated microsystems. Future efforts will be focused on the interface effects, yield, and reliability of the proposed devices based on CNT-Core Cu-TSVs.

## Figures and Tables

**Table 1:** Comparisons of the highest temperatures of each component in the TSV-based filters.

Components	Max. Temp. in Cu-Pillar-TSV Case (°C)	Max. Temp. in BCB-Core-TSV Case (°C)	Max. Temp. in CNT-Core-TSV Case with 20 μm Core Diameter (°C)	Max. Temp. in CNT-Core-TSV Case with 30 μm Core Diameter (°C)	Max. Temp. in CNT-Core-TSV Case with 40 μm Core Diameter (°C)
L1	91.1	94.0	87.8	86.0	85.1
C1	90.2	113.5	86.9	84.6	84.3
L2	90.4	95.2	87.2	85.3	83.9
C2	92.3	114.9	89.1	86.5	85.3
L3	91.2	94.2	88.0	86.4	84.4
C3	89.8	114.7	86.3	85.5	83.6

## Data Availability

The original contributions presented in this study are included in the article. Further inquiries can be directed to the corresponding author.
